# Oxalic acid-mediated phosphorus availability regulates the variations of karst soil organic carbon fractions under drought

**DOI:** 10.3389/fpls.2026.1786025

**Published:** 2026-05-15

**Authors:** Daxiang Ye, Ke Wu, Yueming Liang, Shu Zhang, Xue Wu, Liangran Deng, Fujing Pan, Chun Tu

**Affiliations:** 1 Guangxi Key Laboratory of Environmental Pollution Control Theory and Technology/College of Environmental and Engineering, Guilin University of Technology, Guilin, China; 2 University Engineering Research Center of Watershed Protection and Green Development of Guangxi, Guilin University of Technology, Guilin, China; 3 Key Laboratory of Carbon Emission and Pollutant Collaborative Control, Education Department of Guangxi Zhuang Autonomous Region, Guilin University of Technology, Guilin, China; 4 Karst Dynamics Laboratory of Ministry of Natural Resources, Institute of Karst Geology, Chinese Academy of Geological Sciences, Guilin, China

**Keywords:** drought, karst ecosystem, organic carbon, phosphorus availability, root exudates

## Abstract

Soil organic carbon (SOC) pool in terrestrial ecosystems is increasingly threatened by drought and altered root exudates input under global climate change, but mechanisms changing SOC fractions [Dissolved organic C (DOC), Particulate organic C (POC), Mineral-associated organic C (MAOC), Microbial biomass C (MBC)] in fragile karst ecosystems remain unclear. This study investigated drought (100%, 70%, and 35% field capacity) and the types and input concentrations of exudates (glucose, glycine, and oxalic acid) on SOC fractions through a 90-day incubation experiment with karst shrubland soils from southwest China. The results showed that DOC accumulated associating with reduced microbial and enzymatic activities under drought, while MAOC remained stable. The input of exudates resulted in a reduction in DOC, while simultaneously increasing the activities of β-glucosidase and leucine aminopeptidase under drought. Additionally, the changes in the POC, MBC, total SOC, and the enzyme activities resulting from the input of oxalic acid were slightly more obvious than those caused by glucose and glycine. Random forest models showed that the POC, MAOC, and total SOC were significantly impacted by drought time, available phosphorus (AP), and microbial biomass, while DOC was significantly affected by MBC and βG activity. Additionally, the structural equation models indicated that AP might be a crucial influencing factor in the changes of SOC. This is because of AP was influenced by the drought intensity and time and the exudates input, which in turn constrained microbial and enzyme activities and ultimately affected the changes in karst soil organic C fractions. These results enhanced the understanding of the changes in soil organic C and suggested that when implementing ecological restoration projects, intercropping species with drought tolerance, deep roots, and high oxalic acid exudation into plant communities can be beneficial for the ecological restoration and soil organic C sequestration of karst ecosystems.

## 1 Introduction

Karst regions cover approximately 15% of the world’s land surface (Yuan, 2001; Li et al., 2021). In China, these geological formations are predominantly distributed in the southwest, especially in Guangxi, Guizhou, and Yunnan provinces, encompassing an area of 540,000 Km^2^ and representing one of the largest and most representative karst ecosystems globally (Yuan, 2001). These regions are primarily composed of carbonate rocks, including limestone and dolomite. Due to dissolution processes, a soil–rock dual structure is formed, which is characterized by shallow and highly heterogeneous soils, fissures, caves, and so on (Zhang et al., 2023). The geological drought frequently occurs in these ecosystems. Under the backdrop of global climate change, the increasing frequency and intensity of drought events have emerged as significant threats to fragile karst ecosystems (Jiang et al., 2025; Tang et al., 2025). For example, drought directly affects soil organic carbon (C) accumulation, transformation, and stability by altering plant photosynthate allocation, microbial decomposition processes, and soil physicochemical properties (Deng et al., 2021).

Soil organic C is the largest terrestrial C pool and plays an important role in maintaining soil fertility, microbial function, and climate regulation through its cycle (Scharlemann et al., 2014; Ding et al., 2023), thereby making it a key indicator of ecosystem stability (Beillouin et al., 2023; Díaz-Martínez et al., 2024). Soil organic C fractions are commonly categorized into labile and recalcitrant pools (Yadav et al., 2025). Labile organic C fractions, including dissolved organic C (DOC), microbial biomass C (MBC), and particulate organic C (POC), are characterized by rapid turnover rates, usually ranging from days to years, and act as easily energy sources for soil microorganisms (Li et al., 2018). DOC represents the most labile fraction of organic C and plays a critical role in nutrient translocation by serving as a key substrate for microbial metabolism (LaBrie et al., 2020). MBC serves as an indicator of active organic matter and is essential for assessing soil quality and nutrient dynamics in terrestrial ecosystems (Chen et al., 2023). Additionally, POC is composed of partially decomposed plant residues, including litter and root debris, as well as microbial necromass (Ridgeway et al., 2022). In comparison, recalcitrant fraction such as mineral-associated organic carbon (MAOC) exhibit turnover times longer than 100 years (Zhou et al., 2024), primarily due to stabilization through chemical interactions with soil minerals and physical protection within soil aggregates (Yao et al., 2026).

Soil organic C fractions are impacted by microbial activity under drought. Drought restrains the decomposition of DOC and POC by reducing microbial activity and enzymatic processes, leading to the accumulation of these fractions (Yang et al., 2025). In contrast, MAOC exhibits a strong bonding with calcium, iron, aluminum, etc. Its high chemical stability enables it to have a high resistance to drought and microbial degradation (Lin et al., 2025). However, a study has reported a decline in MAOC during drought in a non-karst ecosystem, which was attributed to alterations in microbial communities and soil aggregation (Su et al., 2020). Conversely, karst ecosystems, characterized by unique geological features including high calcium carbonate content, high pH, and heterogeneous soil distribution, may exhibit fundamentally different changes in soil organic C when exposed to drought (Yang et al., 2025).

Root exudates, which account for approximately 5% to 27% of total photosynthate production (Haichar et al., 2014; Vives-Peris et al., 2020) and are photosynthetic products mediating the interactions among plants, soils, and microorganisms (Salem et al., 2022; Sorty et al., 2025), can influence the transformation of soil organic C fractions. First, exudates make a direct contribution to soil organic C. Exudates act as labile C substrates, which directly contribute to soil C sequestration. They achieve this by modulating soil physicochemical properties through mechanisms such as chelation and pH regulation (Pan et al., 2016), or by promoting soil aggregate stability and facilitating mineral-phase adsorption (Panchal et al., 2022). Second, exudates have a direct impact on microbial activity (Chai and Schachtman, 2022). For instance, the increased exudation of organic acids and sugars can stimulate microbial and enzymatic activities though enhancing nutrient solubilization and acquisition (Williams and Vries, 2020). Alternatively, these increased inputs can enhance microbial necromass, resulting in a notable rise in the quantity and proportion (58.1%–77.9%) of MAOC (Feng et al., 2025). However, under severe drought conditions, the input of exudates into the soil may be constrained by C limitation, as plants prioritize aboveground growth and stress tolerance over C allocation to the rhizosphere (Gargallo-Garriga et al., 2018).

Root exudates consist of low-molecular-weight organics (such as sugars, amino acids, and organic acids) and high-molecular-weight organics (including mucilage and proteins). These exudates, particularly sugars, amino acids, and organic acids, play crucial roles in regulating soil organic C changes (He et al., 2023). Sugars and amino acids are the preferentially utilizable C sources for microbes, and microbial growth thereby influencing changes in soil organic C (Ma et al., 2024). However, the impact of oxalic acid on organic C changes may be greater than that of sugars and amino acids in karst ecosystem. A study has shown that many plants have higher exudate rates of oxalic acid in this ecosystem (Pan et al., 2016). As an important C source for microbes, the dual carboxyl groups of oxalic acid enable direct molecular interactions with soil organic matter, thus actively influencing the turnover dynamics of SOC. Additionally, in karst ecosystem, phosphorus (P) emerges as a pivotal limiting factor for both plant and microbial growth (Pan et al., 2015; Zhang et al., 2015; Chen et al., 2019). Oxalic acid can increase the availability of P (Pan et al., 2016), which promote plant and microbial growth, ultimately changing soil organic C fractions (Hu and Lan, 2020; Wang et al., 2023b; Lan et al., 2025). Nevertheless, in the context of global climate change, the current understanding of how root exudates (such as low-molecular-weight organic acids, sugars, and amino acids) influence changes of soil organic C fractions in karst forest ecosystems under drought remains limited.

To answer the above questions, this study adopted an experimental approach. Soil samples were collected from karst shrublands, which represent a successional stage and are widely distributed across the karst areas of southwestern China, demonstrating high ecological representativeness (Pan et al., 2016). Three drought gradients were established. Simultaneously, three representative root exudates (glucose, glycine, and oxalic acid) were selected at three input levels (no input, low concentration, and high concentration). A total of 441 samples were subjected to a 90-day incubation experiment, and 3,087 subsamples were collected over seven sampling time points. The concentrations of DOC, MBC, POC, MAOC, total SOC, AP, and microbial biomass nitrogen (MBN) were analyzed, as well as the activities of β-glucosidase (βG), β-1,4-N-acetylglucosaminidase (NAG), and leucine aminopeptidase (LAP). The objectives were as follows: (1) to examine the effects of drought and exudates on soil organic C fractions and enzyme activities; (2) to investigate the relationships among these C fractions, microbial biomass, enzyme activities, and AP, and to identify the key influence factors; and (3) to explore the underlying mechanisms how drought and exudates impact changes in soil organic C. Based on the unique characteristics of karst ecosystems, this study proposes three hypotheses: (1) DOC and POC may increase under drought, whereas MAOC remain relatively stable. (2) Among root exudates, oxalic acid may have a greater influence on soil organic C fractions than glucose or amino acids. (3) P availability may be an important factor influencing the changes of karst soil organic C fractions.

## 2 Materials and methods

### 2.1 Soil sample collection

The soil sample was collected from a karst ecosystem situated in the Lijiang River Basin in Guilin, Guangxi Zhuang Autonomous Region, southwestern China (24°55′–25°19′N, 110°37′–110°51′E). This site is characterized by a typical subtropical monsoon climate, with a mean annual temperature of approximately 19 °C and mean annual precipitation ranging from 1800 to 2000 mm, more than 65% of which occurs from May to September (Ning et al., 2023; Pan et al., 2024a). According to the FAO-UNESCO Soil Classification System, the soil type is classified as lithosols (limestone soil), as indicated by our previous studies (Pan et al., 2024a, Pan et al., 2024b).

Previous study has indicated that shrubland represents the most widely distributed vegetation succession stage (stages including grassland, shrubland, shrub-tree forest, and arbor forest) in the karst regions of southwestern China (Pan et al., 2016). During the shrubland stage, five plots (20 m × 20 m) were established (Wu et al., 2025). In November 2022, soil samples (0–20 cm depth) from five sampling points evenly distributed in each plot were collected using stainless steel shovels. The samples from the five plots were thoroughly homogenized to obtain approximately 80 kg of composite soil. This composite soil was then processed by removing plant roots, stones, and other visible debris, air-dried, and passed through a 2-mm sieve.

### 2.2 Experiment

#### 2.2.1 Establishment of drought gradients

To investigate the effects of drought on soil organic C fractions, three drought gradients were established: control (CK, 100% field capacity), mild drought (70% field capacity), and severe drought (35% field capacity) (Liu et al., 2023). Air-dried soil samples, each weighing 100 g, were placed into 250 mL brown wide-mouth bottles. The initial moisture content of the air-dried soil was 8%. The soil field capacity in shrubland was determined to be approximately 26%. As a result, soil moisture levels for CK, mild drought, and severe drought treatments were set at 26%, 18%, and 10%, respectively. To achieve the experimental moisture levels of the three drought treatments, 24.33 mL, 12.33 mL, and 1.04 mL of deionized water were initially added, respectively. A 10-day preliminary experiment was conducted prior to the main experiment. Each sample container was sealed with a cap to minimize water loss through evaporation. This procedure indicated that the average daily evaporation loss was approximately 50 μL. Therefore, to maintain consistent soil moisture conditions throughout the drought experiment, each sample was supplemented with 50 μL of solution daily.

#### 2.2.2 Establishment of exudate levels

To investigate the effects of root exudates on soil organic C fractions, three common root exudates (glucose, glycine, and oxalic acid) were selected. Three experimental treatments were established based on *in-situ* measured root C exudate rates as follows: no input, low concentration (1×, equivalent to the natural exudate rate), and high concentration (3×, three times the natural exudate rate) (Wang et al., 2021).

According to our previous study, the exudate rate of oxalic acid from karst shrub species was measured to be 0.4413 mg/g root/d (Qin, 2023). In the shrubland, the fine root biomass was 2.6417 mg/cm^3^, and the soil bulk density was 1.14 g/cm^3^. For each experimental replicate, 100 g of soil corresponded to a volume of 87.7192 cm^3^ (calculated as100 g/1.14 g/cm^3^). The daily input of oxalic acid per replicate was 0.1023 mg [0.4413 mg/g root/d × (87.7192 cm^3^ × 2.6417 mg/cm^3^)], representing 0.0273 mg C/d [0.1023 mg × (24.00/90.03)]. Based on equivalent C inputs, the daily input of glucose was 0.0683 mg ((180.16/72.06) × 0.0273 mg), and glycine input was 0.0854 mg ((75.07/24.02) × 0.0273 mg). These values are in correspondence with the level of low concentration. The high-concentration treatment was established at three times these amounts, resulting in daily inputs of 0.3069 mg oxalic acid, 0.2049 mg glucose, and 0.2562 mg glycine per replicate.

#### 2.2.3 Preparation of exudate solutions

For low-concentration inputs, 0.2046 g oxalic acid, 0.1366 g glucose, and 0.1708 g glycine were each separately dissolved in deionized water to prepare 100 mL of individual solutions. This procedure ensures that each 50 μL solution contains precisely 0.1023 mg oxalic acid, 0.0683 mg glucose, and 0.0854 mg glycine, respectively. For high-concentration inputs, 0.6160 g oxalic acid, 0.4108 g glucose, and 0.5136 g glycine were individually dissolved to prepare 100 mL solutions, ensuring that a 50 μL solution contains 0.3069 mg oxalic acid, 0.2049 mg glucose, and 0.2562 mg glycine. These solutions were stored at 4 °C and utilized within three days to prevent the degradation and oxidation of these exudates.

#### 2.2.4 Implementation of the incubation experiment

**Figure 1:**
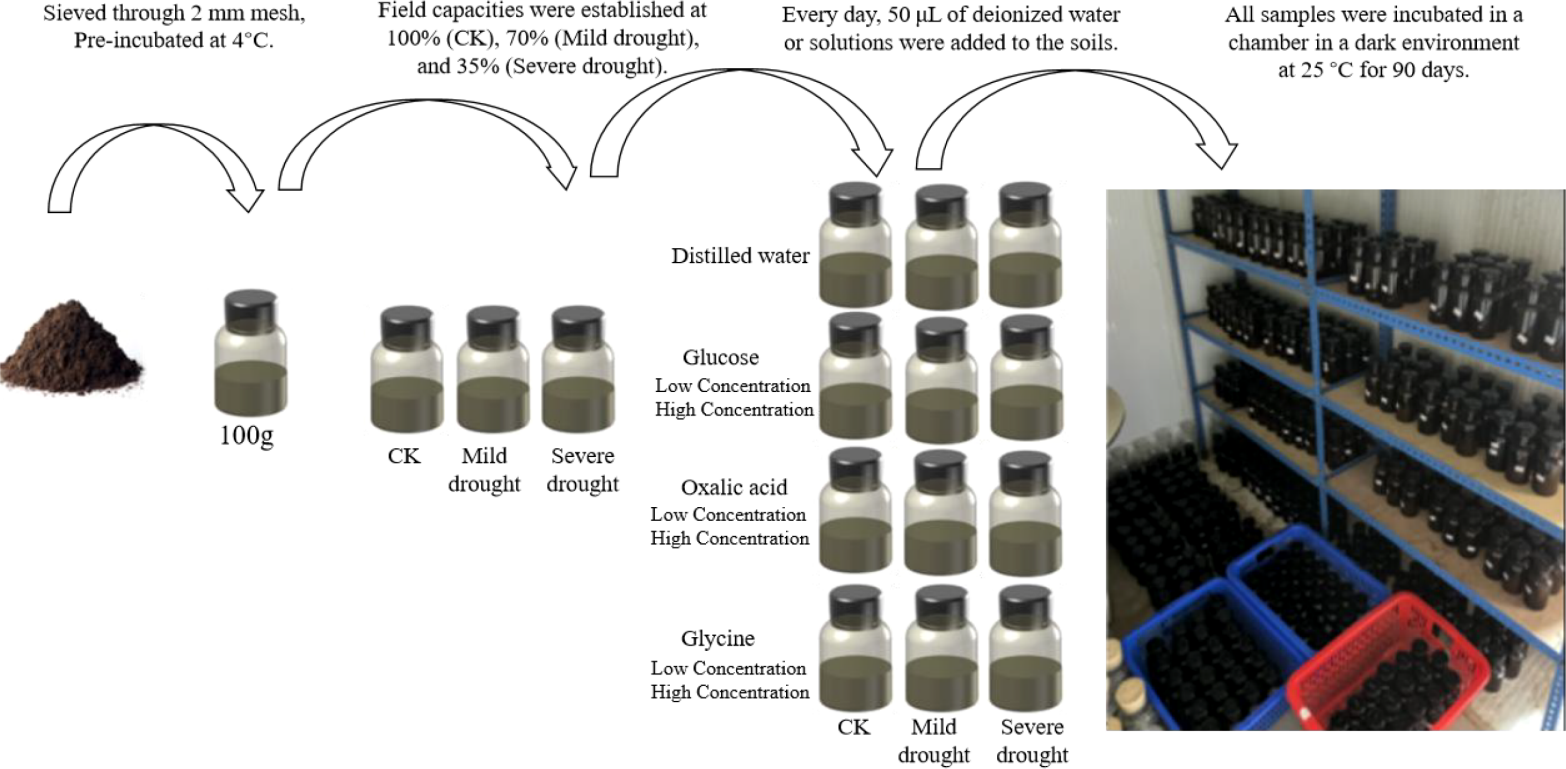
The indoor incubation experiment.

Each treatment was performed in triplicate. A total of 441 samples, comprising 378 experimental samples (3 exudate types × 2 input concentration levels × 3 drought treatments × 7 sampling time points × 3 replicates) and 63 control samples (CK: 3 drought treatments × 7 sampling time points × 3 replicates), were prepared. Every day, 50 μL of deionized water or solutions containing low and high concentration of glucose, oxalic acid, or glycine were added to the corresponding replicates. All samples were incubated in a chamber in a dark environment at 25 °C for 90 days (**Figure 1**). Sampling was carried out on days 1, 4, 7, 15, 30, 60, and 90, resulting in a total of 3,087 subsamples.

### 2.3 Analyses

#### 2.3.1 Separation of soil organic C fractions

The wet sieving method was used to separate soil organic C fractions (Cotrufo et al., 2019). A 10.0 g subsample was transferred into a 150 mL Erlenmeyer flask, and then 50 mL 5 g·L^-1^ sodium hexametaphosphate solution was added. The flask was sealed with aluminum foil and secured with a rubber band to prevent leakage during agitation. The mixture was manually shaken for 3 min, followed by incubation in a reciprocating shaker at 180 r·min^-1^ for 18 hours at 25 °C. The resulting soil suspension was passed through a 53 μm sieve. The residue retained on the sieve was thoroughly rinsed with deionized water using a gentle stream until the filtrate became clear and free of fine soil particles, which typically required 200–1000 mL of water. This part, which is designated as POC (>53 μm), was oven-dried at 60 °C. Subsequently, the suspension was also oven-dried at 60 °C, and this portion of soil is classified as MAOC (<53 μm). Ultimately, all dried samples were ground to pass through a 100-mesh sieve and were stored.

#### 2.3.2 Determination of organic C fractions and other parameters

The total SOC, POC, and MAOC concentrations were measured by wet oxidation using KCr_2_O_7_ + H_2_SO_4_ and titration with FeSO_4_. DOC were extracted using 0.5 mol·L^−1^ K_2_SO_4_ and quantified with a total organic C analyzer (Shimadzu TOC-Vwp; Shimadzu Corporation, Kyoto, Japan). MBC and MBN were assessed using the chloroform fumigation-extraction method, in which both fumigated and unfumigated soil samples were extracted with 0.5 mol·L^−1^ K_2_SO_4_ (Wu et al., 1990). MBC was measured like DOC, while MBN was analyzed using a FIAstar 5000 flow injection analyzer (FOSS, Hillerød, Denmark). AP was extracted with NaHCO_3_ and determined by colorimetric analysis based on the molybdenum blue method. The activities of βG, NAG, and LAP were measured by fluorescence spectrophotometry (Finzi et al., 2006; Wu et al., 2025). The acetate buffer (50 mM, pH 5) was employed to prepare the sample suspension for measuring βG, NAG, and LAP activities. The substrates for these activities are 4-methylumbelliferyl-β-D-glucoside, 4-methylumbelliferyl-N-acetyl-β-D-glucosaminide, and L-leucine-7-amido-4-methylcoumarin hydrochloride, respectively. The sample suspensions were prepared by homogenizing 1 g of soil with 125 mL of acetate buffer. Then, twelve 200-µL suspensions of each sample were dispensed into 96-well microplates, and were evenly divided into blank, negative control, and quench standard groups. These microplates were incubated at 20 °C for 4 h in the dark. Subsequently, a 10-µL aliquot of 1.0 M NaOH was added to each well to stop the reaction. Ultimately, the activities of three enzymes were measured by a microplate fluorometer (Infinite M200 PRO; TECAN, Switzerland) equipped with 365 nm excitation and 450 nm emission filters.

### 2.4 Statistical analyses

The normality and homoscedasticity of data were evaluated before conducting statistical analyses. The 90-day temporal changes of the MAOC, POC, DOC, MBC, and total SOC and the βG, NAG, LAP activities under drought with exudates input were visualized using line graphs. Additionally, a four-way analysis of variance (ANOVA) was conducted to evaluate the main and interactive effects of drought gradients, drought time, exudate types, and exudate input concentrations on these organic C fractions and enzyme activities. Pearson correlation analysis was employed to examine the relationships among the MAOC, POC, DOC, MBC, total SOC, MBN, and AP concentrations and the βG, NAG, LAP activities. Furthermore, random forest models (“randomForest” package in R 4.4.0, R Core Team, 2025) were applied to identify key factors that influence the variations of POC, MAOC, DOC, and total SOC. Finally, structural equation model (SEM) was used to evaluate the influence path of drought and exudates on POC, MAOC, DOC, and total SOC.

## 3 Results

### 3.1 Drought and exudate input impacted soil organic C fractions

**Figure 2:**
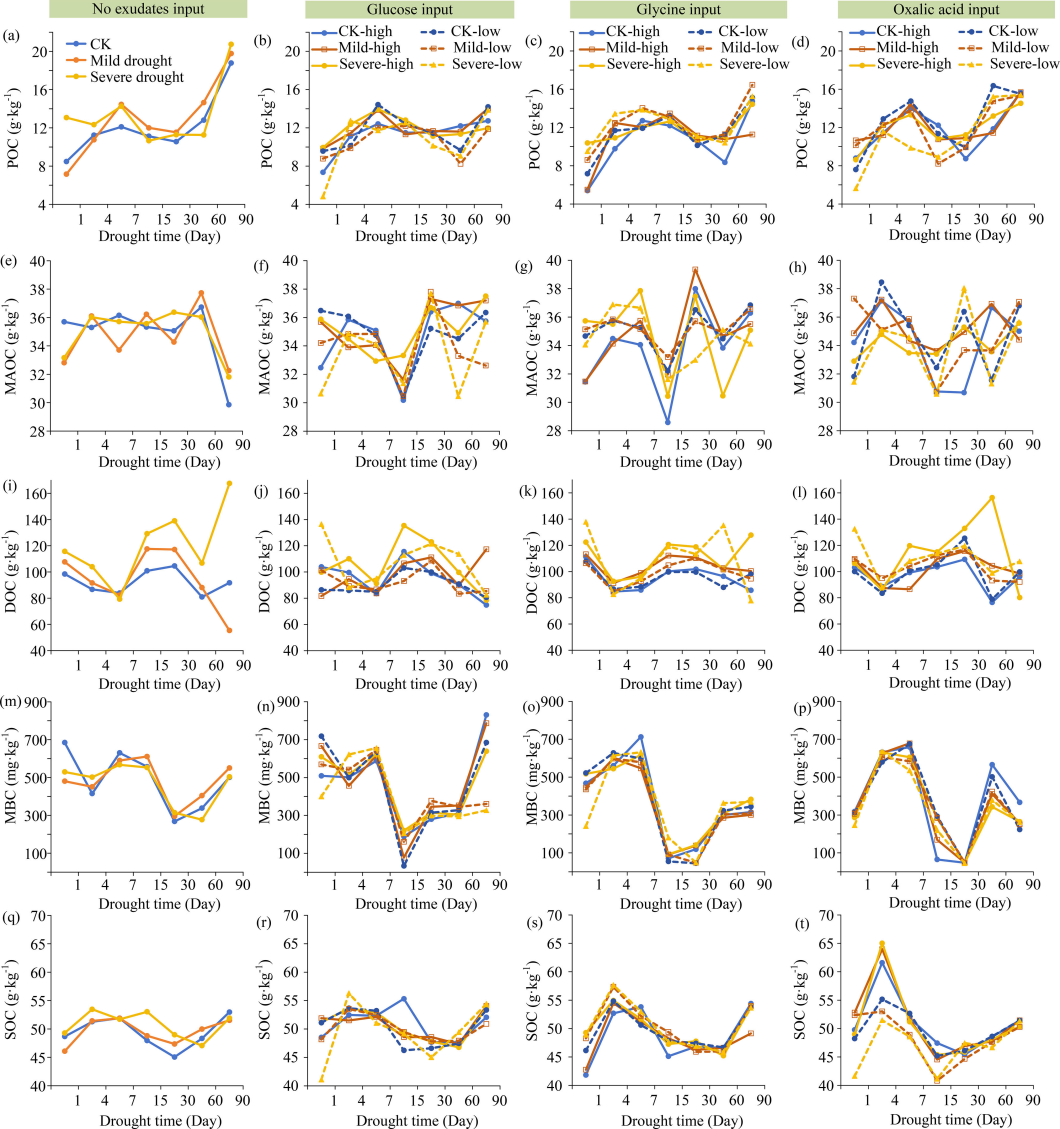
Drought and exudates input impacted soil organic C fractions. CK, Wet; Mild, Mild drought; Severe, Severe drought. Low, Low concentration input of exudates; High, High concentration input of exudates. The same below. **(a)** The no exudetes inout on POC; **(b)** The glucose inout on POC; **(c)** The glycine inout on POC; **(d)** The oxalic acid inout on POC; **(e)** The no exudetes inout on MAOC; **(f)** The glucose inout on MAOC; **(g)** The glycine inout on MAOC; **(h)** The oxalic acid inout on MAOC; **(i)** The no exudetes inout on DOC; **(j)** The glucose inout on DOC; **(k)** The glycine inout on DOC; **(l)** The oxalic acid inout on DOC; **(m)** The no exudetes inout on MBC; **(n)** The glucose inout on MBC; **(o)** The glycine inout on MBC; **(p)** The oxalic acid inout on MBC; **(q)** The no exudetes inout on total SOC; **(r)** The glucose inout on total SOC; **(s)** The glycine inout on total SOC; **(t)** The oxalic acid inout on total SOC.

**Figure 3:**
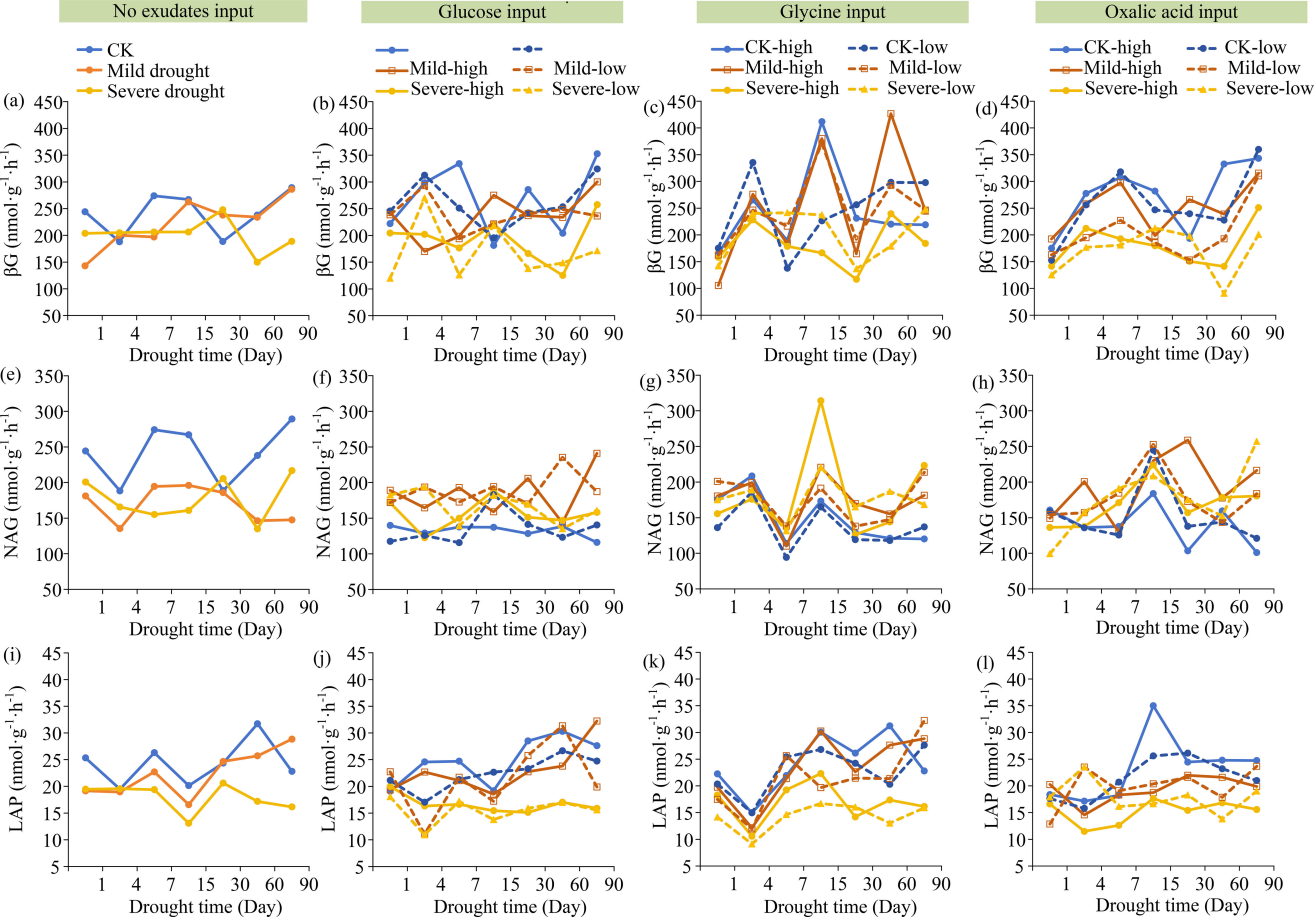
Drought and exudates input impacted soil enzyme activities. **(a)** The no exudetes inout on βG activity; **(b)** The glucose inout on βG activity; **(c)** The glycine inout on βG activity; **(d)** The oxalic acid inout on βG activity; **(e)** The no exudetes inout on NAG activity; **(f)** The glucose inout on NAG activity; **(g)** The glycine inout on NAG activity; **(h)** The oxalic acid inout on NAG activity; **(i)** The no exudetes inout on LAP activity; **(j)** The glucose inout on LAP activity; **(k)** The glycine inout on LAP activity; **(l)** The oxalic acid inout on LAP activity.

DOC increased under drought compared to wet (CK), while MAOC exhibited slight variations. The input of glucose, glycine, and oxalic acid resulted in lower DOC levels compared to the condition with no exudates input. POC increased with the increasing glucose concentration and soil moisture, but it decreased as glycine increased. MBC, MAOC, and total SOC had no significant differences between different types or input concentrations of exudates. MBC was positively correlated with both the input concentrations of exudates and soil moisture. Additionally, oxalic acid had a slightly greater impact on POC, MAOC, DOC, MBC, and SOC than glucose and glycine (**Figure 2**). The βG and LAP activities increased with higher soil moisture and exudates input, whereas NAG activity showed an inverse trend, except in the condition with no exudates input (**Figure 3**).

The ANOVA analysis showed that the drought time exerted a significant influence on all five organic C fractions and three enzyme activities. Drought gradients had a notable influence on DOC and three enzyme activities. Exudate types had a significant influence on total SOC, POC, and MBC. Exudate input concentrations significantly influenced total SOC and LAP activity. The drought time had the highest influence on these parameters, followed by drought gradients and exudate types, whereas exudate input concentrations had the least influence. Consequently, the interaction effects among the drought time, drought gradients, and exudate types had significant impacts on the five organic C fractions and three enzyme activities (**Table 1**).

**Table 1:** The individual and interactive effects of drought, exudates input, and drought time on soil organic C fractions and enzyme activities.

Factors	Total SOC		MAOC		POC		DOC		MBC		βG		NAG		LAP	
	F	*p*	F	*p*	F	*p*	F	*p*	F	*p*	F	*p*	F	*p*	F	*p*
Drought gradients	0.31	0.736	0.99	0.373	0.55	0.213	15.56	**<0.001**	2.93	0.055	40.18	**<0.001**	26.51	**<0.001**	137.8	**<0.001**
Exudate types	3.76	**0.024**	0.20	0.818	3.70	**0.026**	1.64	0.195	55.79	**<0.001**	0.39	0.678	1.02	0.363	0.95	0.388
Exudate concentrations	10.43	**0.001**	0.45	0.504	0.19	0.666	0.39	0.533	3.15	0.077	2.27	0.133	0.22	0.644	9.61	**0.002**
Drought time	96.29	**<0.001**	16.53	**<0.001**	93.44	**<0.001**	7.77	**<0.001**	241.4	**<0.001**	11.54	**<0.001**	7.42	**<0.001**	21.69	**<0.001**
Gradients × Types	3.21	**0.013**	0.62	0.650	2.86	0.024	0.06	0.993	0.97	0.424	1.62	0.169	1.46	0.215	0.78	0.540
Gradients × Concentrations	2.26	0.106	3.21	**0.042**	4.21	**0.016**	0.05	0.954	1.54	0.216	0.44	0.648	0.40	0.670	0.29	0.748
Gradients× Time	1.29	0.227	1.54	0.109	1.21	0.276	0.60	0.841	3.98	**<0.001**	1.93	**0.030**	1.11	0.350	7.39	**<0.001**
Types × Concentrations	20.67	**<0.001**	3.62	**0.028**	7.95	**<0.001**	0.11	0.900	2.07	0.128	1.99	0.138	0.61	0.542	5.83	**0.003**
Types × Time	9.85	**<0.001**	1.70	0.066	6.94	**<0.001**	0.67	0.783	34.84	**<0.001**	5.77	**<0.001**	2.43	**0.005**	8.02	**<0.001**
Concentrations × Time	1.72	0.116	1.73	0.114	1.28	0.267	0.43	0.856	6.69	**<0.001**	0.65	0.688	0.89	0.505	2.28	**0.037**
Gradients × Types × Concentrations	1.26	0.287	0.21	0.931	1.25	0.290	0.23	0.922	0.45	0.770	0.84	0.498	0.31	0.875	0.92	0.452
Gradients × Types × Time	2.20	**0.001**	1.79	**0.014**	1.74	**0.019**	0.35	0.999	2.59	**<0.001**	2.32	**0.001**	1.07	0.375	1.48	0.071
Gradients × Concentrations × Time	2.24	**0.010**	1.60	0.090	1.23	0.264	0.50	0.912	2.71	**0.002**	1.08	0.377	1.21	0.278	1.48	0.131
Types × Concentrations × Time	6.78	**<0.001**	3.93	**<0.001**	3.04	**<0.001**	0.57	0.864	7.49	**<0.001**	1.73	**0.060**	1.66	0.076	5.28	**<0.001**
Gradients × Types × Concentrations × Time	1.75	**0.018**	1.36	0.124	1.27	0.186	0.60	0.935	2.51	**<0.001**	1.30	0.163	1.11	0.328	2.30	**0.001**

Bold values indicated the significant influence of the factors.

### 3.2 Correlations between organic C fractions and other parameters

**Figure 4:**
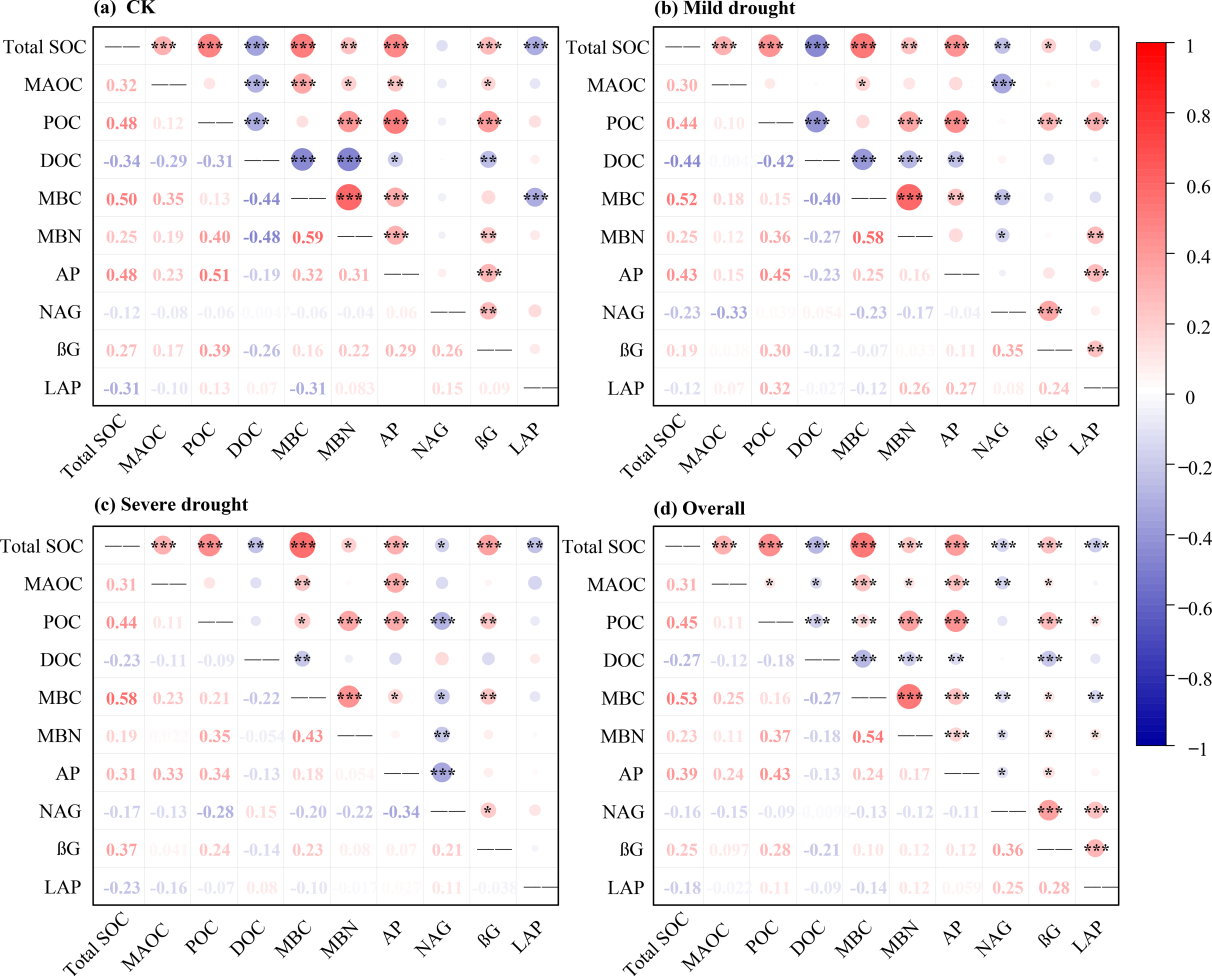
Correlation between soil organic C fractions, AP, and enzyme activities under different drought gradients.

Under the wet condition (CK), total SOC was positively correlated with MAOC, POC, MBC, MBN, AP, and βG activity, but was negatively correlated with DOC and LAP activity. MAOC showed a significant positive correlation with MBC, MBN, AP, and βG activity but a negative correlation with DOC. POC was positively related to MBN, AP, and βG activity, but was negatively related to DOC. DOC was negatively correlated with MBC, MBN, AP, and βG activity (**Figure 4a**).

Under the mild drought condition, total SOC was positively correlated with MAOC, POC, MBC, MBN, AP, and βG activity, but was negatively correlated with DOC and NAG activity. MAOC showed a significant positive correlation with MBC but a negative correlation with NAG activity. POC was positively related to MBN, AP, and βG and LAP activities, but was negatively related to DOC. DOC was negatively correlated with MBC, MBN, and AP (**Figure 4b**).

Under the severe drought condition, total SOC was positively correlated with MAOC, POC, MBC, MBN, AP, and βG activity, but was negatively correlated with DOC and NAG and LAP activities. MAOC showed a significant positive correlation with MBC and AP. POC was positively related to MBC, MBN, AP, and βG activity, but was negatively related to NAG activity. DOC was negatively correlated with MBC (**Figure 4c**).

Under the overall condition, total SOC was positively correlated with MAOC, POC, MBC, MBN, AP, and βG activity, but was negatively correlated with DOC and NAG and LAP activity. MAOC showed a significant positive correlation with POC, MBC, MBN, AP, and βG activity but a negative correlation with DOC and NAG activity. POC was positively related to MBC, MBN, AP, and βG and LAP activities, but was negatively related to DOC. DOC was negatively correlated with MBC, MBN, AP, and βG activity (**Figure 4d**).

**Figure 5:**
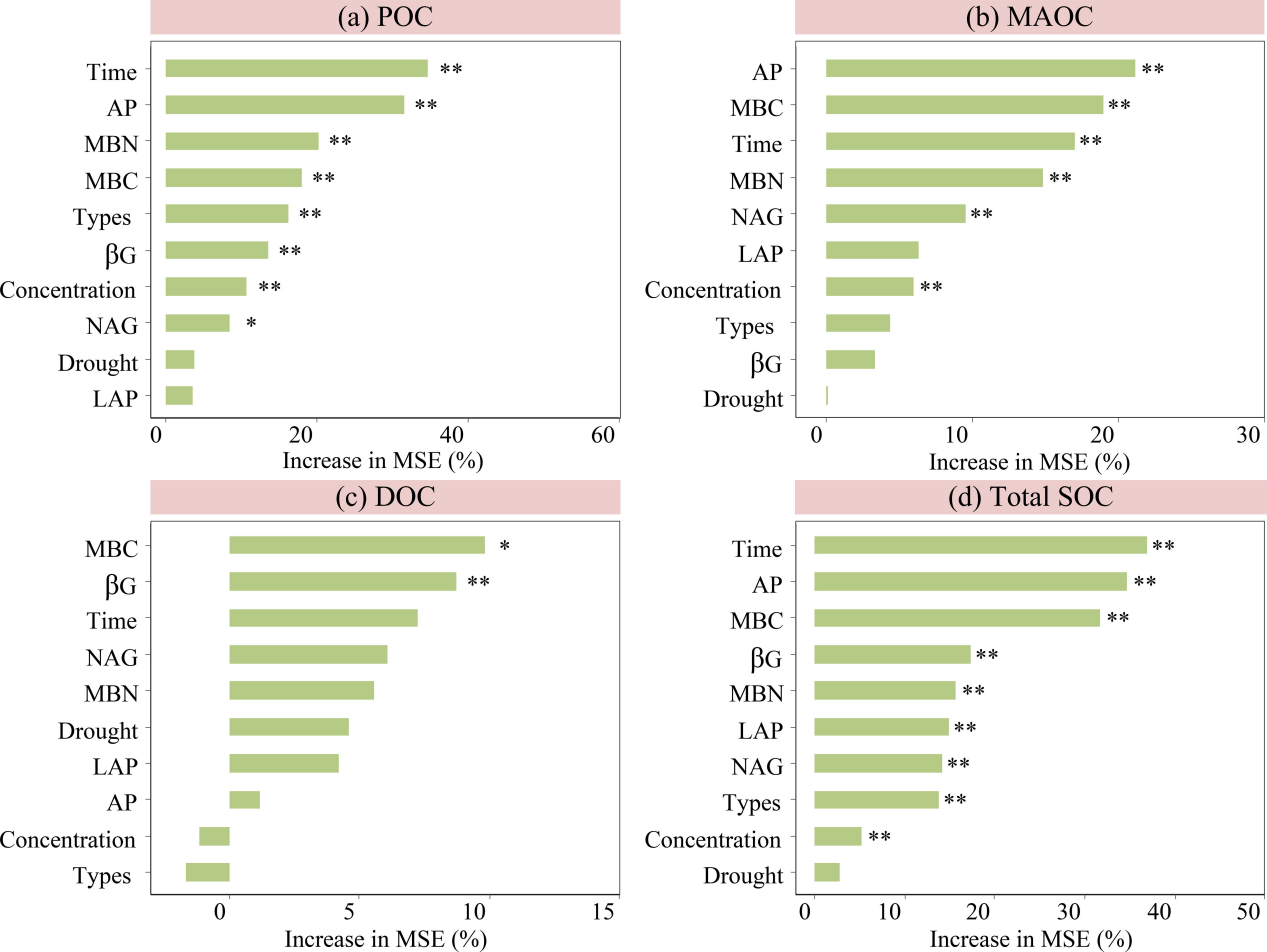
Ranking of the important factors affecting POC, MAOC, DOC, and total SOC. Drought, drought gradients; Time, drought time; Types, exudate types; Concentration, exudate input concentrations.

The random forest model results showed that POC was significantly impacted by the important factor drought time, followed by the AP, MBN, MBC, exudate types, βG activity, exudate input concentrations, and NAG activity (**Figure 5a**). MAOC was significantly affected by AP, followed by the MBC, drought time, MBN, NAG activity, and exudate input concentrations (**Figure 5b**). DOC was mainly influenced by MBC, followed by the βG activity (**Figure 5c**). The total SOC was significantly affected by drought time, followed by the AP, MBC, βG activity, MBN, LAP activity, NAG activity, and the types and input concentrations of exudates (**Figure 5d**). Thus, the changes of POC, MAOC, and total SOC were strongly associated with drought time, AP, microbial biomass, and enzyme activities (**Figures 5a, b, d**), while DOC was significantly linked to MBC and βG activity (**Figure 5c**).

### 3.3 The pathways affecting organic C fractions

**Figure 6:**
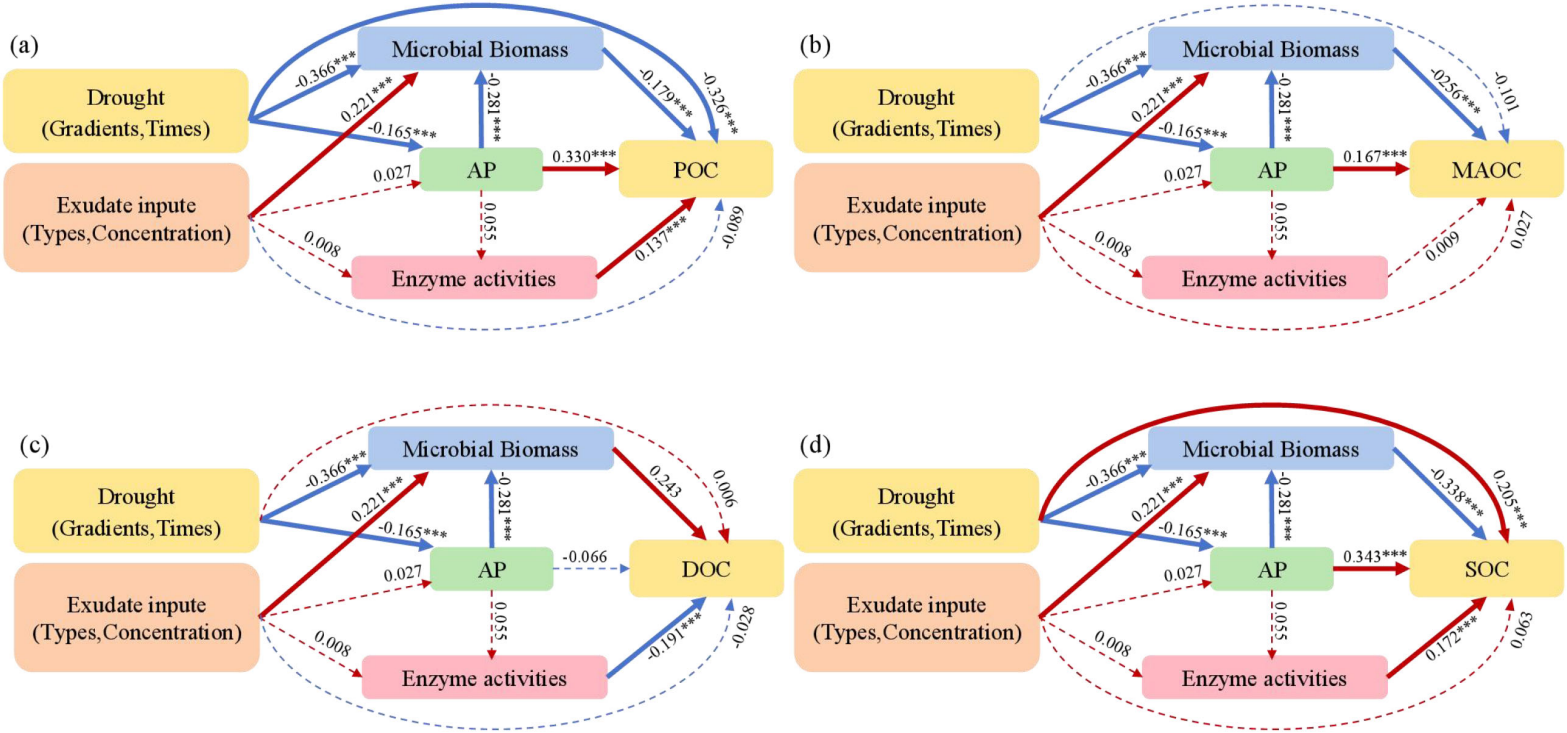
The influence path of drought and exudate on POC **(a)**, MAOC **(b)**, DOC **(c)**, and total SOC **(d)** by the structural equation model. The parameters of these models: **(a)** X_2_ = 5.889, degrees of freedom = 3, n = 441, CFI = 0.996, AGFI = 0.969, *p* = 0.117, RMSEA = 0.047; **(b)** X_2_ = 5.889, degrees of freedom = 3, n = 441, CFI = 0.996, AGFI = 0.969, *p* = 0.117, RMSEA = 0.047; **(c)** X_2_ = 5.889, degrees of freedom = 3, n = 441, CFI = 0.996, AGFI = 0.969, *p* = 0.117, RMSEA = 0.047; **(d)** X_2_ = 5.889, degrees of freedom = 3, n = 441, CFI = 0.996, AGFI = 0.969, *p* = 0.117, RMSEA = 0.047. Red represents positive influence, blue represents negative influence. -–, *p* > 0.05, *p < 0.05, **p < 0.01, ***p < 0.001.

Structural equation model results (**Figure 6**; **Supplementary Material S2**) showed that drought gradients and times negatively impacted microbial biomass and AP, and AP negatively impacted microbial biomass. However, Exudates input positively influenced microbial biomass. AP and enzyme activities positively affected POC, MAOC, and total SOC, but negatively affected DOC. However, the effects of microbial biomass were contrary to those of AP and enzyme activities.

## 4 Discussion

### 4.1 Effects of drought and exudates input on karst soil organic C fractions

The present study showed that DOC increased under drought, while MAOC remained relatively stable (**Figure 2**). The different C fractions exhibited distinct responses to drought. Under drought conditions, the metabolic activities and growth of microbes, as well as the activities of extracellular enzymes (*i.e.* β-glucosidase, β-1,4-N-acetylglucosaminidase) (**Figures 2**, **3**), are generally constrained. This can be attributed to the reduction of soil moisture and the limitation of water availability (Bogati and Walczak, 2022; Oram et al., 2025). These reductions will decrease the decomposition and mineralization rate of SOC, leading to an increase in DOC (Shi et al., 2024). Thus, drought was the major environmental stressor that significantly affected the changes of soil organic C, which supported the first hypothesis.

DOC, as the most microbially labile C fraction, is influenced by the delicate balance between its solubility and the rate of microbial assimilation. First, microbial respiration and associated physiological processes are inhibited under drought, leading to reduced C utilization efficiency, lower nutrient uptake capacity, and decreased consumption and mineralization of DOC (Deng et al., 2021). Second, the diffusion of DOC is constrained under this condition, thereby limiting its accessibility to microorganisms. Third, drought inhibits extracellular enzymatic activities, significantly decreasing both the synthesis and degradation of DOC (Zhang et al., 2022). The negative correlation between β-glucosidase activity and DOC in this study verified this situation (**Figure 4**). Therefore, DOC was primarily influenced by MBC and β-glucosidase activity (**Figure 5**).

The input of exudates decreased DOC but increased β-glucosidase and leucine aminopeptidase activities (**Figures 2**, **3**). These exudates encompass a wide variety of types, among which low-molecular-weight organics such as glucose, amino acids, and organic acids have direct and rapid effects. For example, glucose can enhance microbial activity because it serves as a C source for microorganisms (Islam et al., 2023). Glycine, with a relatively high nitrogen content, can alleviate microbial nitrogen limitation and boost enzymes production (Allison and Jastrow, 2006). Oxalic acid, serving as both a C source and a nutrient mobilizer, can supply energy for microbes and promote the decomposition of organic matter (Clarholm et al., 2015). Consequently, the input of these exudates facilitated the transformation of DOC under drought (**Figure 2**). Moreover, the changes in the POC, MBC, total SOC, and NAG and LAP activities resulting from the input of oxalic acid were slightly more obvious than those caused by glucose and glycine (**Figures 2**, **3**). Thus, oxalic acid might have a slightly greater influence on soil organic C fractions than glucose or amino acids, which predominantly supported the second hypothesis.

Although MAOC was affected by AP, MBC, drought time, MBN, NAG activity, and exudate input concentrations (**Figure 5**), it still appeared relatively stable responding to drought (**Figure 2**). This phenomenon can be explained by the following two main reasons. (1) Under drought conditions, the decrease in soil pore water limits the contact between microorganisms and organic C in aggregates, thereby inhibiting C decomposition (Thotakuri et al., 2025). (2) Calcium can form stable complexes with soil organic C in karst ecosystems (Wang et al., 2025b). In these ecosystems, soils are characterized by high calcium content, which then form the stable “calcium bridge” with organic C. This unique stabilization mechanism can largely protect soil organic C from environmental changes. Therefore, MAOC was largely unaffected by drought. These results showed that the changes of MAOC are primarily influenced by the mineral protection mechanism, highlighting the important role of the unique geological background on soil organic C stabilization in karst ecosystems. In conclusion, the labile soil C fractions are primarily impacted by microbial and enzymatic activities, while the recalcitrant C fraction is predominantly influenced by the mineral protection mechanism in karst ecosystems.

### 4.2 Factors affected soil organic C fractions

This study showed that the POC, MAOC, and total SOC were strongly impacted by drought time, AP, microbial biomass, and enzyme activities (**Figure 5**). Although the variations of POC, MAOC, and total SOC were relatively slight under drought, prolonged drought times had a more significant impact on these organic C parameters. Drought reduce soil phosphorus availability and microbial and enzymatic activities, ultimately impedes the transformation of POC and MAOC and the stabilization of total SOC (Wang et al., 2025a). This was supported by the results of structural equation models in this study (**Figure 6**). Therefore, drought time is the primary factor affecting these organic C parameters. Nevertheless, this impact remained relatively minor when compared to that of the DOC.

DOC exhibited a negative correlation with MBC and βG activity (**Figures 4c**, **5c**). It is well established that drought can reduce microbial and enzymatic activities, resulting in increasing DOC. Nevertheless, the increasing soil moisture and exudate input concentrations may mitigate the adverse effects of drought. This study revealed that the activities of β-glucosidase and leucine aminopeptidase increased with the increasing soil moisture and exudate input concentrations. Under this condition, the heightened activity of β-glucosidase can facilitate the dissolution of cellulose substrates (Lu et al., 2024), thus increasing the C supply for microbes. Similarly, the increase in leucine aminopeptidase activity reflects an increase in nitrogen sources to meet microbial demand (Bao et al., 2024). These patterns highlighted the critical role of soil moisture and exudate input concentrations in soil organic C transformation by regulating microbial and enzymatic activities (Sun et al., 2024). Additionally, the elevated moisture levels alleviate the diffusion constraints of soil enzymes, ultimately changing the transformation of DOC. In contrast, the activity of β-1,4-N-acetylglucosaminidase reduced with the increasing input of exudates (**Figure 3**). This pattern suggested a decreased microbial dependence on recalcitrant nitrogen sources (such as chitin and peptidoglycan degradation) when labile nutrients are readily accessible (Xu et al., 2021).

The random forest models showed that available phosphorus is a highly ranked factor influencing the variations of POC, MAOC, DOC, and total SOC (**Figures 5**, **6**). This result highlighted the function of phosphorus limitation in modulating soil organic C cycle in karst ecosystems (Clarholm et al., 2015; Pan et al., 2026). In these ecosystems, which are characterized by high calcium and pH levels, phosphorus has a tendency to form complexes with calcium or become embedded within organic matter, resulting in phosphorus limitation for both plants and microbes (Pan et al., 2024b). An increase in soil available phosphorus can alleviate this limitation in plants, stimulating greater exudation of root exudates and thereby enhancing mineral nutrient exchange between plants and microbes (Wu et al., 2025). Moreover, the improvement in phosphorus availability alleviates microbial phosphorus limitation (Li et al., 2025), promotes microbial biomass accumulation, and enhances the production of extracellular enzymes (Pan et al., 2018). These processes facilitate the decomposition and transformation of soil organic C, including the conversion of DOC into POC and MAOC, as well as the transformation of POC into MAOC. Thus, phosphorus indirectly regulates the changes of soil organic C by modulating microbial activity, reflecting a tightly coupled phosphorus-carbon interaction in karst ecosystems, and corroborating the third hypothesis.

### 4.3 Implications of drought and exudates input for ecosystem restoration and management

The findings of this study provide three key insights regarding the restoration and management of soil organic C in karst ecosystems. First, the patterns of DOC accumulation and MAOC stability under drought suggest that moderate drought may enhance soil organic C sequestration. Drought would reduce microbial and enzyme activities, which decrease the mineralization of labile organic C and ultimately maintain the stability of soil organic C pool. However, it should be noted that the input of a large amount of root exudates can promote the improvement of microbial and enzymatic activities, and ultimately may alleviate the impact of drought on soil organic C changes. Meanwhile, it is also necessary to recognize that root exudates are the important sources of soil organic C. The input of a large amount of root exudates into soils can improve soil organic C sequestration and facilitate soil aggregate formation. Our previous study has found that some plant species in karst ecosystems, such as *Pteroceltis tatarinowii* and *Quercus glauca*, exhibit high oxalic acid exudation from roots (Pan et al., 2016). Therefore, the configuration of more such plants in the restoring vegetations may greatly contribute to the stabilization and sequestration of soil organic C in karst ecosystems.

Secondly, available phosphorus, might serve as a key factor impacting soil organic C changes in karst ecosystems (**Figure 5**). Phosphorus limitation in karst ecosystems can be alleviated by enhanced interactions between plant roots and soil microorganisms (Wu et al., 2025). On one hand, plants can improve phosphorus availability by solubilizing insoluble phosphorus minerals through rhizosphere acidification (Pan et al., 2026). On the other hand, mycorrhizal fungi, such as arbuscular mycorrhizae, establish symbiotic associations with plant roots. Their hyphal networks penetrate into soils, thereby enhancing root absorption capacity for phosphorus and promoting phosphorus availability through the increased activities of enzymes such as phosphatases and phytases (Liang et al., 2016). Therefore, the collaborative plant-microbe strategy can be utilized to alleviate phosphorus limitation, activate extracellular enzyme system, and accelerate the transformation of soil organic C fractions.

Ultimately, microbial biomass, enzyme activity, and DOC demonstrated remarkable sensitivity to drought stress (**Figures 5**, **6**), indicating the importance of prioritizing deep-rooted plant species such as *Quercus glauca* in ecological restoration projects (Pan et al., 2026). Their well-developed root systems facilitate access to deep soil water, thus maintaining rhizosphere moisture and creating favorable conditions for the survival and reproduction of microbes. Moreover, integrating nitrogen-fixing species such as *Platyosprion platycarpum* and *Amorpha fruticosa* into the vegetation community can boost overall plant biomass and improve the ecological stoichiometry of plant litter (Wang et al., 2023a; Pan et al., 2026). This restoration approach may increase the input of plant-derived C into soils and provide sustained support for the stabilization and expansion of soil organic C pool in karst ecosystems. However, the efficacy of this method requires further validation through more extensive empirical research.

## 5 Conclusions

The mechanisms by which drought and exudates alter SOC fractions in fragile karst ecosystems remain unclear. A 90-day incubation experiment was conducted to investigate the effects of three exudates (glucose, glycine, oxalic acid) at three input concentrations on SOC fractions under three drought gradients. The key findings were as follows: (1) Soil DOC and MBC responded rapidly and significantly to drought and exudates input, whereas MAOC demonstrated relatively high resistance to these disturbances. DOC accumulated under drought due to the constrained microbial decomposition. Oxalic acid might exert a more influence on the changes of soil organic C than glucose or amino acids. (2) The interactions of drought intensity, drought time, exudate types, and available phosphorus greatly influenced the changes of soil organic C in karst ecosystems. These findings indicated that plant species with drought tolerance, high oxalic acid exudation, and well-developed deep root systems may facilitate soil organic C sequestration and stabilization in karst ecosystems. Future research should focus on: (1) Simulation of the effects of diverse plant functional types on soil organic C sequestration and stabilization under different drought patterns in restoring vegetations of karst ecosystems; (2) Long-term monitoring of the mechanisms underlying the interaction between drought stress and root exudates and their influence on soil organic C sequestration and stabilization in the field.

## Data Availability

The datasets presented in this article are not readily available because No. Requests to access the datasets should be directed to panfujing@glut.edu.cn.
