# Supplementary material for: Oxalic acid-mediated phosphorus availability regulates the variations of karst soil organic carbon fractions under drought
Source: Front Plant Sci. 2026 May 15;17:1786025. doi: 10.3389/fpls.2026.1786025 (PMC13219385; doi:10.3389/fpls.2026.1786025)
Supplement: Supplementary file 1 [file SupplementaryFile1.docx]

Fig.S1 Effects of drought and exudates input on soil microbial biomass nitrogen (MBN) and available phosphorus (AP).

Fig.S2 The direct and indirect effects of drought and exudates on POC (a), MAOC (b), DOC (c), and total SOC (d) by the structural equation model.
